# Prognostic Significance of Overexpression of BCL9 and TPX2 in High-Grade Clear Cell Renal Cell Carcinoma: Prognostic Markers for Metastasis and Survival

**DOI:** 10.3390/ijms26094114

**Published:** 2025-04-26

**Authors:** Michał Kasperczak, Iga Kołodziejczak-Guglas, Filip Kasperczak, Maciej Wiznerowicz, Andrzej Antczak

**Affiliations:** 1 Department of Urology, J. Struś Hospital in Poznań, Szwajcarska 3, 61-285 Poznan, Poland; filiperkasperczako@gmail.com (F.K.); maciej.wiznerowicz@iimo.pl (M.W.); aa26@poczta.onet.pl (A.A.); 2 International Institute for Molecular Oncology, 60-203 Poznan, Poland; iga.kolodziejczak@iimo.pl; 3 University Hospital of Lord’s Transfiguration, 61-848 Poznan, Poland

**Keywords:** clear-cell renal cell carcinoma, biomarkers, prognosis, BCL9, TPX2

## Abstract

Clear-cell renal cell carcinoma (ccRCC) is a kidney cancer associated with poor prognosis and limited treatment options. Identifying new prognostic markers is crucial. This study investigates the potential of BCL9 and TPX2, two proteins involved in cancer progression, to predict patient outcomes This study analyzed protein abundance data from the CPTAC cohort (110 ccRCC and 84 NAT samples) using LC-MS/MS. BCL9 and TPX2 were validated via immunohistochemistry (IHC) in an independent cohort (52 ccRCC samples). Patients were stratified into high- and low-expression groups based on IHC scores. Survival analyses were conducted, and Reactome pathway enrichment analysis was performed. BCL9 and TPX2 were significantly upregulated in ccRCC compared to NAT. In the validation cohort, high BCL9 levels were associated with shorter progression-free survival (PFS) but not OS, while high TPX2 levels correlated with shorter overall survival (OS) but not PFS. Pathway analysis linked BCL9 to Wnt signaling and TPX2 to cell cycle regulation. Elevated BCL9 and TPX2 are associated with poor prognosis in ccRCC. These proteins are potential prognostic markers and therapeutic targets.

## 1. Introduction

Clear-cell renal cell carcinoma (ccRCC) is diagnosed in approximately 300,000 new patients worldwide each year and accounts for more than 100,000 deaths annually [1]. This subtype represents 70–80% of all renal cell carcinoma (RCC) cases [2]. Although ccRCC can often be detected early and treated effectively, metastasis occurs in up to one-third of cases [3]. Despite advances in novel therapies, ccRCC remains associated with a poor prognosis, with an overall five-year survival rate of only 10%, making it the most lethal form of kidney cancer [4].

Histologically, ccRCC originates from proximal tubule cells in the kidney [5]. As the disease progresses, it commonly metastasizes to the lungs, liver, and bones [6]. Notably, the clear-cell subtype of renal cancer is defined by distinct chromosomal and genetic alterations. Chromosome 3p loss is observed in more than 90% of sporadic ccRCC cases, leading to the loss of one copy of four key tumor suppressor genes: *VHL*, *PBRM1*, *BAP1*, and *SETD2* [7]. Additionally, the remaining alleles of these genes are frequently mutated in ccRCC [8].

To date, the somatic loss or mutation of *VHL*, a gene that regulates cellular oxygen sensing, is considered the key initiating event in ccRCC development [3,9]. *VHL* mutations lead to the stabilization of hypoxia-inducible transcription factors HIF-1a and HIF-2a, resulting in transactivation of their target genes, including vascular endothelial factor (VEGF), erythropoietin (EPO), and transforming growth factor (TGF). Consequently, this drives cell proliferation, angiogenesis, and metastasis [10].

Mutations in chromatin modifiers such as *PBRM1* and *SETD2* contribute to the global deregulation of the epigenetic landscape in ccRCC. *PBRM1* is essential for the stability of the SWI/SNF chromatin remodeling complex, which plays a critical role in enhancing chromatin accessibility [11,12]. In contrast, SETD2 catalyzes H3K36me3 deposition, preventing cryptic transcription initiation and enabling DNA methylation within gene bodies [13]. Given these multi-dimensional alterations in cellular metabolism and DNA organization, ccRCCs often exhibit chemotherapy resistance [14]. These challenges highlight the urgent need to identify novel therapeutic targets and reliable prognostic factors, not only to enhance treatment efficacy but also to enable earlier detection of specific RCC subtypes.

B-cell CLL/lymphoma 9 (BCL9) is a coactivator of the Wnt/β-catenin signaling pathway. By enhancing β-catenin-mediated transcription, BCL9 promotes transcription of genes involved in cell proliferation and metastasis [15]. In the intestinal epithelium, Wnt signaling drives regeneration by inducing the differentiation of crypt stem cells [16]. Consequently, aberrant Wnt signaling plays a direct role in the pathogenesis of colorectal carcinoma [17]. However, deregulated Wnt signaling is implicated in the progression of various cancers, contributing to the maintenance of a stem-like phenotype and poor clinical outcomes [18]. In the context of ccRCC, BCL9 has not been sufficiently explored yet.

Tumorigenesis is also driven by chromosomal mis-segregation during mitosis, which leads to aneuploidy and chromosomal instability [19,20]. Targeting protein for Xenopus kinesin-like protein 2 (TPX2), a microtubule-associated protein, facilitates mitotic spindle assembly [21] by activating a key regulator of cell progression and mitosis, Aurora A kinase [22,23]. Both TPX2 and Aurora A kinase are frequently overexpressed in cancer and are functionally linked to the maintenance of chromosomal instability and aneuploidy [24,25]. TPX2 has already been reported as a key unregulated factor in ccRCC and has been linked to poor patient prognosis and cancer progression [26].

The aim of this study was to identify and validate prognostic protein biomarkers in ccRCC, specifically focusing on TPX2 and BCL9. Through integrative analysis of proteomic data and immunohistochemical validation, we found that elevated levels of these regulatory proteins are linked to cancer progression and poor survival outcomes in ccRCC patients.

## 2. Results

### 2.1. The BCL9 and TPX2 Protein Levels Are Significantly Elevated in ccRCC

**Figure 1:**
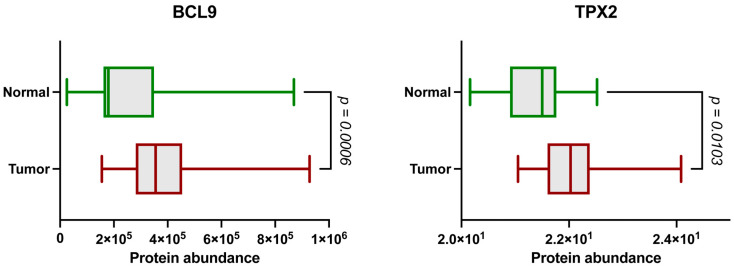
Protein abundance between NATs (green) and tumor samples (red) for BCL9 and TPX2 based on LC-MS/MS. Y-axis indicates log_2_-transformed normalized protein abundance values. Data from the CPTAC cohort (n tumor = 110, n normal = 84). *p*-values obtained by Mann–Whitney U test.

To explore potential protein markers in ccRCC, we analyzed publicly available LC-MS/MS data from the Clinical Proteomic Tumor Analysis Consortium (CPTAC) cohort. BCL9 and TPX2 are significantly upregulated proteins in tumor tissues compared to the normal adjacent tissues (NATs) (Figure 1). Therefore, we selected them for further analysis and validation [27].

### 2.2. Immunohistochemical Analysis of BCL9 and TPX2 Levels on Validation Cohort of ccRCC Patients

**Figure 2:**
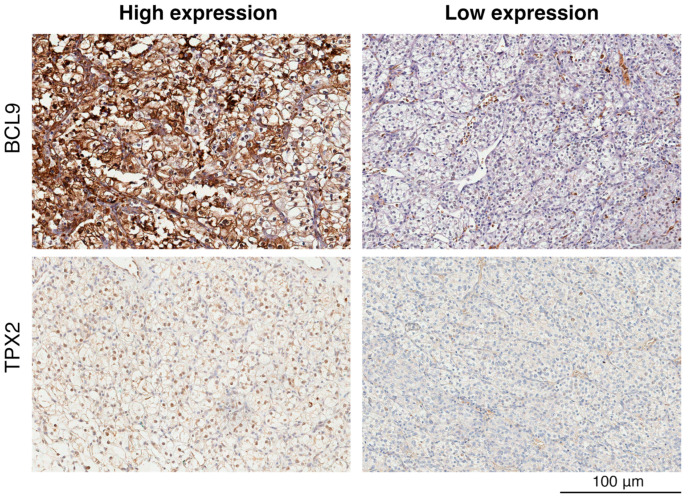
Immunohistochemical analysis exemplifying immunostaining patterns in tumor sections for BCL9 and TPX2 divided into high and low expression levels in the validation cohort. Positive immunoreaction is represented by brown, while negative immunoreaction is shown as blue.

Given our focus on the prognostic potential of BCL9 and TPX2, we assessed their protein abundance through immunohistochemistry. Analysis of 52 ccRCC tissue specimens from the validation cohort revealed significant variability in protein abundance between the samples (Figure 2). Notably, BCL9 exhibited uneven distribution: in some cells, it accumulated in the nucleus and cellular membrane, while in others, it was also observed in the cytoplasm. Although BCL9 has predominantly been characterized as a nuclear protein, evidence suggests its cytoplasmic localization, which may depend on the cell’s differentiation state [28]. In contrast, TPX2 localized primarily in the nucleus.

### 2.3. BCL9 and TPX2 Protein Levels Allow for Distinguishing Two Distinct Groups Depending on IHC Score

**Figure 3:**
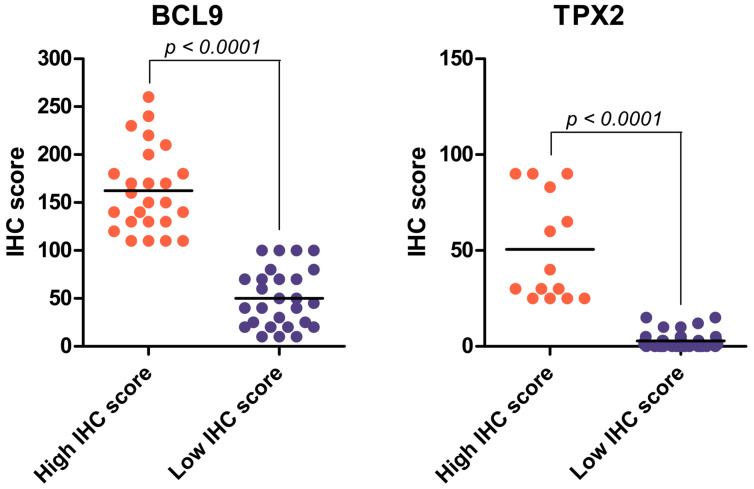
The distribution of IHC scores for BCL9 and TPX2 demonstrates variability between the high score (red) and low score (blue) groups. The difference in IHC scores between the two groups was statistically significant for both proteins (Wilcoxon rank-sum and Mann–Whitney U tests, *p* < 0.0001).

The IHC assay revealed varying levels of BCL9 and TPX2 expression across the samples. After visually inspecting the sections, we quantified the IHC scores. Based on these calculations, we identified two distinct categories for both factors: high and low IHC score groups, with a threshold of 104 for BCL9 and 16 for TPX2 (Figure 3). The difference in IHC scores between the low and high groups was statistically significant for both BCL9 and TPX2. IHC scores for BCL9 exhibited greater variability than those for TPX2, consistent with its uneven distribution across different cells. However, for both proteins, the IHC scores were relatively consistent within each group—particularly for TPX2—regarding their coherent abundance.

### 2.4. High BCL9 and TPX2 Protein Levels Are Associated with Worse Survival in ccRCC Patients

**Figure 4:**
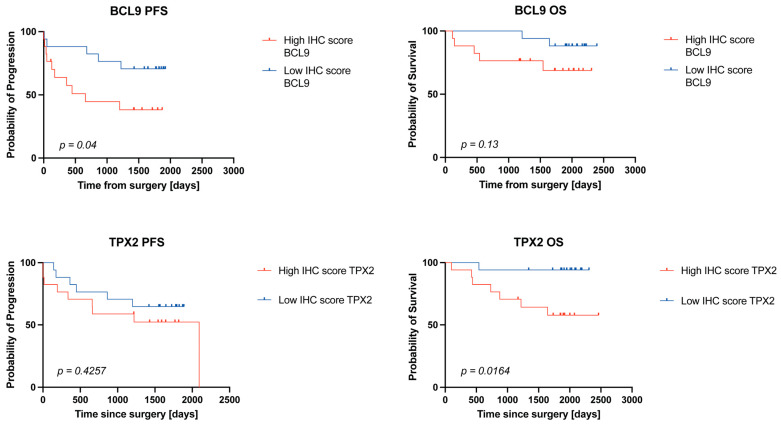
Progression free (PFS) and overall survival (OS) analysis for BCL9 and TPX2 between high and low IHC score groups. *p*-values based on Kaplan–Meier method and the log-rank (Mantel–Cox) test.

To further investigate the prognostic potential of BCL9 and TPX2 in ccRCC, we conducted a survival analysis comparing the previously established high and low IHC score groups. Both overall survival (OS) and progression-free survival (PFS) were assessed (Table 1). The analysis revealed that patients with higher BCL9 protein levels had a significantly lower PFS rate (*p* = 0.04, HR = 2.906), whereas OS was not significantly affected (Figure 4). In contrast, patients with high TPX2 levels exhibited a significantly lower OS rate (*p* = 0.0164, HR = 5.52), while PFS remained unchanged. These findings suggest that elevated levels of both BCL9 and TPX2 are associated with poor prognosis, although their clinical implications differ. Lower BCL9 levels appear to stabilize disease progression without significantly improving overall survival. Conversely, lower TPX2 levels are associated with significantly improved overall survival, despite no observed changes in perceptible cancer-related symptoms.

### 2.5. BCL9 and TPX2 Regulate Wnt Signaling Pathway, Cell Cycle, and Cell Proliferation

**Figure 5:**
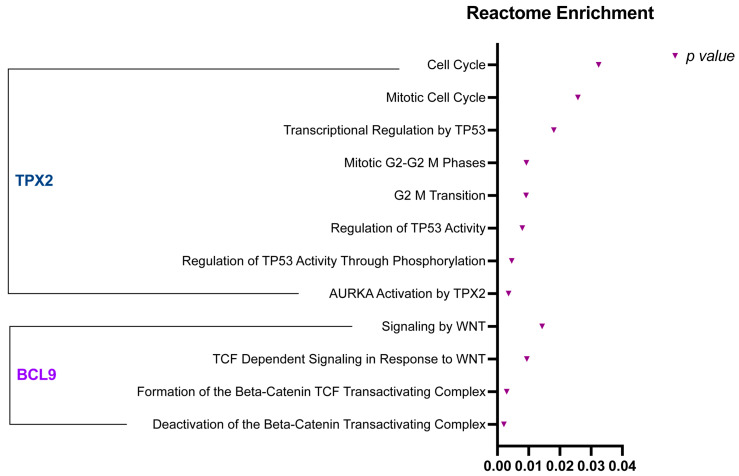
Significant Reactome enrichment pathways for *BCL9* and *TPX2. p*-values of statistically significant (*p*-value < 0.05) enriched terms associated with each protein are represented as purple triangles.

To further explore the roles of BCL9 and TPX2 in ccRCC progression and pathogenesis, we conducted a Reactome pathway enrichment analysis for these two proteins (Figure 5). This approach enables the identification of significant biological processes and molecular pathways associated with specific factors. Our analysis revealed multiple significantly enriched pathways, aligning with the known biological functions of BCL9 and TPX2. The most enriched pathways linked to BCL9 were associated with Wnt signaling, including the formation and deactivation of the β-catenin transactivation complex. In contrast, TPX2 was predominantly involved in cell cycle regulation, particularly the G2-M transition and mitosis. Additionally, the most significantly enriched pathways for TPX2 involved the activation of two key regulators of cell proliferation: Aurora kinase A and TP53.

## 3. Discussion

The treatment of clear-cell renal cell carcinoma remains a significant challenge due to its low survival rate and resistance to chemotherapy. Identifying novel prognostic markers and optimizing treatment strategies are, therefore, crucial. In this study, we provide evidence that elevated levels of regulatory proteins BCL9 and TPX2 are linked to ccRCC progression and poor patient prognosis.

By analyzing LC-MS/MS data from the CPTAC cohort, we found that both BCL9 and TPX2 levels were significantly elevated in ccRCC tumor samples compared to paired normal adjacent tissues. This observation was further validated by immunohistochemistry in an independent cohort of 52 ccRCC patients, stratified into low- and high-expression groups. Our analysis revealed that high BCL9 abundance was significantly associated with longer progression-free survival (PFS), whereas overall survival (OS) remained unchanged. In contrast, high TPX2 expression was correlated with significantly lower OS, while PFS was not statistically affected.

Statistical modeling suggests that the correlation between PFS and OS decreases in diseases with longer post-progression survival [29]. Since PFS does not always predict OS, it is considered a less reliable endpoint for patient benefit assessment. The observed discrepancy between OS and PFS in our study may indicate distinct clinical implications of elevated BCL9 and TPX2 levels. According to this model, TPX2 appears to be a more reliable prognostic factor, and, paradoxically, the lack of correlation between PFS and OS may suggest prolonged survival post-progression [30].

As a key regulator in mitotic spindle assembly, TPX2 has been extensively studied in the context of mitotic fidelity and genome instability in cancer. Conditional ablation of Tpx2 in mouse embryos disrupts microtubule nucleation and spindle formation, leading to mitotic exit without proper chromosome segregation [31]. TPX2 activates Aurora kinase A (AurkA), which localizes to centrosomes and spindle pole microtubules to regulate centrosome maturation, chromosome alignment, and separation [25,32]. Notably, co-overexpression of TPX2 and AurkA exacerbates mitotic defects compared to AurkA overexpression alone, resulting in increased aneuploidy and micronuclei formation in daughter cells [33].

TPX2 has already been identified as a key hub gene in ccRCC. A microarray analysis by Zhang et al. (2019) identified TPX2 among 11 significantly upregulated hub genes in ccRCC, with high TPX2 levels correlating with lower OS rates [34]. This finding was further corroborated in 2020 by three independent research groups analyzing distinct datasets [35,36]. Liu et al. reported a positive correlation between TPX2 expression and ccRCC stage [34], while Xu et al. demonstrated by IHC that elevated TPX2 mRNA levels correspond to high TPX2 protein abundance in ccRCC tissues [36]. TPX2 is also recognized as a potential prognostic and therapeutic marker in the papillary subtype of renal cell carcinoma (PRCC). Wang et al. demonstrated that knock-down of TPX2 in a cellular model of PRCC (SKRC39 cell line) leads to a worse ability of cells to migrate and a significant decrease in cell proliferation, measured by edU incorporation into DNA [37]. Additionally, TPX2 is strongly co-expressed with KIF20A, a factor implicated in the development and progression of many cancer types, including melanoma [38] and cervical squamous cell carcinoma [39]. A recent study has identified KIF20A as a potential prognostic marker in ccRCC, where its elevated expression is associated with increased immune cell infiltration and poor clinical outcomes [40]. Collectively, these studies reinforce TPX2 as a key upregulated factor in ccRCC, strongly linked to poor prognosis and cancer progression.

There is emerging evidence suggesting that elevated TPX2 protein levels may contribute to poor patient prognosis by influencing immune cell infiltration within the tumor microenvironment (TME). Tumor-associated macrophages (TAMs), the most abundant innate immune cells in the TME, play a crucial role in modulating tumor progression and immune response. Unlike normal macrophages, TAMs contribute to tumor progression by enhancing cancer cell proliferation, facilitating metastasis, and suppressing adaptive immune cells within the TME [41]. Wang et al. reported that in high-risk patients with type 2 papillary renal cell carcinoma (PRCC2), elevated TPX2 levels correlate with a significantly higher infiltration of tumor-associated M1 macrophages. Their retrospective analysis further revealed that high-risk PRCC2 patients responded more favorably to everolimus, an mTOR inhibitor, compared to low-risk patients [42]. This association between increased M1 macrophage infiltration and high TPX2 expression was validated in an independent PRCC cohort [43], suggesting that the poor prognosis observed in ccRCC patients with high TPX2 levels might be TAMs-associated.

Further evidence supporting TPX2 as a key marker in kidney cancer development comes from a 2022 study by Peerapen et al. The researchers explored the link between kidney stone disease (KSD) and RCC progression, demonstrating that calcium oxalate monohydrate (COM), a primary crystalline component of KSD, induces carcinogenic traits in normal renal cells. COM exposure led to the downregulation of well-established RCC tumor suppressor genes, including ARID1A, PTEN, and VHL, while significantly increasing TPX2 expression. Additionally, COM crystals contributed to cisplatin resistance, underscoring the need to identify reliable biomarkers for optimizing oncological treatment strategies [44].

TPX2 has been explored as a potential therapeutic target across multiple cancer types. In patient-derived xenograft models of pancreatic cancer, reduced TPX2 expression correlates with increased sensitivity to PARP inhibitor (PARPi) treatment. Furthermore, inhibiting TPX2 S634 phosphorylation with a cell-penetrating peptide enhances pancreatic cancer cell responsiveness to PARPi [45]. In breast cancer, TPX2 overexpression has been linked to heightened sensitivity to a c-Src inhibitor, dasatinib, through activation of the Yes-associated protein 1 (YAP) signaling pathway [46]. BRCA2-deficient cancer exhibits heightened sensitivity to TPX2 and AurkA inactivation through alisertib treatment, which delays mitotic progression [47]. Additionally, in pancreatic ductal adenocarcinoma (PDAC), high TPX2 levels are associated with diminished efficacy of gemcitabine-based chemotherapy, indicating its potential as a predictive marker for gemcitabine resistance [48]. A recent study has highlighted the therapeutic potential of selectively disrupting the interaction between TPX2 and Aurora A kinase. Novel small-molecule inhibitor CAM2602 has been developed to target this protein–protein interaction (PPI), thereby inhibiting mitotic progression and tumor growth. In preclinical models, CAM2602 has shown efficacy by synergizing with paclitaxel treatment, effectively suppressing the outgrowth of pancreatic cancer cells [49]. TPX2 has also been identified as a potential marker of sensitivity to anti-PD-1 therapy, as its elevated expression correlates with increased immune cell infiltration [50]. Consistent with previous findings, TPX2 overexpression enhanced CD8+ T cell-mediated antitumor immunity and improved the efficacy of anti-PD-1 therapy in a hepatocellular carcinoma patient-derived xenograft mouse model [51]. Given its role in chromosomal mis-segregation and mitotic dysregulation, TPX2 may serve as both a promising therapeutic target and a prognostic biomarker for optimizing treatment strategies in ccRCC.

Given the five-year overall survival (OS) rate of 10% in ccRCC, the significant increase in progression-free survival (PFS) associated with lower BCL9 levels represents a meaningful improvement in patient quality of life. BCL9 functions as a coactivator of Wnt/β-catenin-dependent transcription, driving cancer progression through the activation of genes involved in crucial developmental processes [52]. To initiate downstream transcription events, BCL9 recruits the PYGO coactivator to β-catenin/TCF complexes [53]. Direct inhibition of the Wnt/β-catenin pathway has emerged as a promising therapeutic strategy. ST316, a first-in-class antagonist disrupting BCL9/β-catenin interaction, is currently undergoing Phase 1–2 clinical trials (NCT05848739) for patients with advanced solid tumors [54]. Beyond its canonical role in Wnt signaling, BCL9 has recently been shown to interact with paraspeckle proteins in interchromosomal regions [55] and to modulate both direct targets and upstream regulators of STAT3 [56].

STAT3 plays a critical role in inflammation and immunity, therefore implicating BCL9 in the immunoregulation of the TME. However, direct evidence of a BCL9-STAT3 interaction remains limited. In 2019, Feng et al. developed a selective set of inhibitory peptides that disrupt the β-catenin/BCL9 interaction. These inhibitors suppressed cancer cell growth in a mouse colorectal cancer model, promoted cytotoxic T-cell infiltration, and increased dendritic cell abundance within the TME. As a result, BCL9-inhibited cancer cells became more sensitive to PD-1 inhibitors [57]. In a follow-up study, Feng et al. compared pharmacological inhibition of Bcl9 with Bcl9 knockdown (KD) in mouse colon cancer models. They confirmed that Bcl9 KD reduces tumor growth and enhances CD8+ T-cell infiltration. Single-cell RNA sequencing (scRNA-seq) analysis revealed that Bcl9 suppression upregulates CD155 expression, a key immune checkpoint ligand. CD155 interacts with both the costimulatory receptor CD226 and the inhibitory receptor CD96, influencing immune responses ranging from enhanced NK cell adhesion and cytotoxicity to tumor immune evasion and metastasis [58,59]. Targeting the CD155-CD226 axis has emerged as a promising strategy for enhancing the response to anti-PD-1 therapy, as Bcl9-depleted or -inhibited colorectal cancer cells exhibit improved therapeutic sensitivity [60]. These findings align with studies in triple-negative breast cancer, where BCL9 inhibition synergized with PD-1/PD-L1 antibodies, leading to tumor growth suppression [61]. Additionally, BCL9 KD inhibits the proliferation and invasive ability of papillary thyroid carcinoma cells and was identified as a risk factor for neck lymph metastasis [62]. Supporting these results, Zhao et al. demonstrated that BCL9 expression negatively correlates with dendritic cell infiltration in breast cancer, suggesting a broader role for BCL9 inhibition in shaping a favorable immune microenvironment across multiple cancer types [63]. 

In addition to its role in shaping the tumor immune microenvironment—particularly by promoting CD8+ T-cell and dendritic cell infiltration—BCL9 has also been shown to influence endothelial cell fate in a mouse model of colon cancer. Wei et al. performed scRNA-seq on Bcl9-depleted or Bcl9-inhibited tumor samples, revealing that Bcl9 inhibition suppresses endothelial cell differentiation into extracellular matrix cells, thereby impairing angiogenesis and tumor vascularization [64]. Using a similar experimental approach, the authors further demonstrated that Bcl9 inhibition disrupts macrophage polarization from M0 to M2, a process critical for tumor-associated immunosuppression. As a result, Bcl9 depletion was suggested to attenuate inflammation driven by M0 and M1 macrophages while preventing the formation of tumor-promoting M2 macrophages [65].

On the other hand, BCL9 has not been extensively studied in the context of ccRCC. The only study to date conducted in renal cancer identified BCL9 as a direct target of microRNA-218 (miR-218). Transfection of miR-218 mimics into ccRCC cells leads to a reduction in BCL9 expression at both the mRNA and protein levels, whereas transfection of miR-218 inhibitors has the opposite effect. Additionally, miR-218 overexpression suppresses ccRCC cell proliferation, as assessed by MTT assay [66]. Further research is required to determine whether BCL9-associated changes in the tumor microenvironment are relevant to ccRCC. However, given existing evidence, BCL9 inhibition may represent a promising therapeutic strategy to enhance tumor sensitivity to anti-PD-1 treatment. This could be particularly beneficial for advanced-stage RCC patients, where treatment options such as nephrectomy are no longer viable [67].

The primary limitation of this study is the small validation cohort used for immunohistochemistry, which, in consequence, might limit the universality of the findings. To fully evaluate the prognostic value of TPX2 and BCL9 in ccRCC, their levels should be validated in a larger and more diverse patient cohort. Given that TPX2 has already been established as a clinically relevant factor in renal cancer, further validation would be particularly valuable for BCL9, which emerges as a potential target for enhancing tumor sensitivity to anti-PD-1/L1 therapy. Notably, in this study, we do not perform any functional studies that could directly confirm the implications of elevated BCL9 and TPX2 levels in ccRCC progression. Additionally, further research is needed to investigate the interplay between TPX2 and BCL9 in renal cancer progression, particularly in the context of their role in modulating the tumor immune microenvironment.

## 4. Materials and Methods

### 4.1. Patients and Cohorts

#### 4.1.1. Publicly Available CPTAC Discovery Cohort

In this study, we analyzed a ccRCC discovery cohort from the Clinical Proteomic Tumor Analysis Consortium (CPTAC), consisting of 110 untreated cases and 84 paired normal adjacent tissue (NAT) samples, as previously described by Clark et al. [27]. To identify key protein markers in ccRCC, we integrated publicly available liquid chromatography–tandem mass spectrometry (LC-MS/MS) protein abundance data with a comprehensive literature review. Based on their biological relevance and significantly higher abundance in tumor samples, we selected two proteins, BCL9 and TPX2, for further investigation. The protein levels of these markers were subsequently validated on a separate cohort using immunohistochemistry (IHC).

#### 4.1.2. IHC Validation Cohort

The validation cohort included 52 ccRCC samples from individuals aged 31 to 84, of whom 23 were also part of the CPTAC discovery cohort. Detailed demographic and clinicopathological data (age, sex, race, tumor grade, and stage) were collected for all patients (Supplementary Table S1). Over a five-year follow-up, 26 patients developed metastases, while the remaining 26 did not. Only adult patients with histopathologically confirmed ccRCC were included. Ethical approval was obtained in accordance with CPTAC guidelines. Patients who had received systemic treatment or had been diagnosed with other cancers within the previous 12 months were excluded from the study.

### 4.2. Immunohistochemistry and Pathology Evaluation

Immunohistochemical (IHC) staining was performed using the Dako Auto-stainer Link 48 with preprogrammed staining protocols and the EnVision visualization kit (Cat. No. K800221-2, Dako, Agilent Technologies Inc., Carpinteria, CA, USA). Tissue samples were retrospectively collected, fixed in 10% neutral buffered formalin for 24 h, and subsequently embedded in paraffin. Four-micron sections were cut from the formalin-fixed paraffin-embedded (FFPE) blocks and mounted on positively charged slides.

Antigen retrieval was performed using Dako Target Retrieval Solution, High pH (Cat. No. S2367, Dako, Agilent Technologies Inc., Carpinteria, CA, USA), in a PT Link Pre-Treatment Module (Dako, Agilent Technologies Inc., Carpinteria, CA, USA) at 97 °C for 20 min. Following antigen retrieval, sections were incubated with primary antibodies for 30 min at room temperature.

IHC-stained slides were independently assessed by three pathologists using the H-score method. This semi-quantitative approach incorporates both staining intensity and the percentage of positive cells, yielding a score from 0 to 300. The H-score was calculated using the following equation:

H-score = (% of cells stained at intensity 1 × 1) + (% of cells stained at intensity 2 × 2) + (% of cells stained at intensity 3 × 3)

where:

- Intensity 1 indicates weak staining.
- Intensity 2 indicates moderate staining.
- Intensity 3 indicates intense staining.

The H-score reflects the overall staining intensity and distribution within a specimen, providing a comprehensive assessment of protein abundance.

52 ccRCC FFPE samples were analyzed for BCL9 and TPX2 protein levels using IHC. Samples were categorized into two groups based on their H-scores: patients with scores above the cutoff were classified as the “High IHC score” group, while those with scores below the cutoff were assigned to the “Low IHC score” group (Table 2). The cutoff value for each protein was determined as the average H-score across all 52 IHC slides.

### 4.3. Digital Image Acquisition and Archiving

A ScanScope AT Turbo whole-slide scanner (Aperio/Leica Microsystems, Vista, CA, USA) digitized all IHC slides at 20× magnification. The resulting digital images, saved in .svs format, were analyzed using ImageScope software (version 12.3.3, Aperio, Vista, CA, USA) for detailed pathological assessment. Access to these digitized images was securely managed via a password-protected Synology RackStation server (RS18017xs+, Synology Inc., Taipei, Taiwan).

### 4.4. Statistical Analysis

Protein abundance differences between normal and tumor tissues in ccRCC were analyzed using the non-parametric Wilcoxon rank-sum test. The Wilcoxon rank-sum test and the Mann–Whitney U test were applied for comparisons of IHC scores between the high and low IHC score groups in the validation cohort. Overall survival (OS) and progression-free survival (PFS) analyses were conducted by stratifying the validation cohort into high and low IHC score groups for each protein target. Survival outcomes were compared using the Kaplan–Meier method and the log-rank (Mantel–Cox) test. Hazard ratios (HR) with 95% confidence intervals (CI) were calculated to assess the impact of protein expression on survival. All statistical analyses were performed using GraphPad Prism 10 (GraphPad Software Inc., Boston, MA, USA, with a *p*-value < 0.05 considered statistically significant.

### 4.5. Reactome Pathway Enrichment Analysis

To gain deeper insights into the biological processes and tumor mechanisms associated with BCL9 and TPX2, we performed a Gene Ontology (GO) enrichment analysis. This analysis aimed to identify each protein’s most significantly enriched Reactome pathways, providing insight into their functional roles in ccRCC.

GO enrichment analysis for BCL9 and TPX2 was conducted using Enrichr (https://maayanlab.cloud/Enrichr accessed on 20 December 2024), an interactive web-based tool for gene list enrichment analysis. GO terms were assessed within the Biological Process (BP) category. Enrichr calculates enrichment by combining statistical significance from Fisher’s exact test with multiple hypothesis correction. The results include GO terms ranked by adjusted *p*-values and combined scores, facilitating the identification of overrepresented biological processes linked to the input genes. A significance threshold of *p* < 0.05 was applied to ensure biological relevance.

## 5. Conclusions

This study highlights the potential utility of TPX2 and BCL9 protein levels as prognostic markers in ccRCC. High TPX2 abundance correlated with poor overall survival, while elevated BCL9 levels were associated with shorter progression-free survival. Since OS is a more reliable indicator of patient outcomes, TPX2 emerges as the stronger biomarker candidate. However, both proteins hold promise for patient stratification and targeted therapeutic strategies, making them valuable candidates for further clinical investigation.

## Figures and Tables

**Table 1:** Impact of protein expression measured by IHC on patients’ survival in ccRCC validation cohort. HR and 95% CIs for *BCL9* and *TPX2*, evaluating their impact on PFS and OS.

	Progression-Free Survival (PFS)		Overall Survival (OS)	
	Hazard Ratio (HR)	95% CI of HR	Hazard Ratio (HR)	95% CI of HR
BLC9	2.906	1.030–8.198	3.158	0.7030–14.19
TPX2	1.534	0.5352–4.398	5.52	1.368–22.27

**Table 2:** Classification of IHC specimens based on H-scores.

Protein	H-Score Range—High IHC Score Group	H-Score Range—Low IHC Score Group	Reaction
BCL9	120–280	10–80	Nuclear
TPX2	25–220	0–15	Cytoplasmic, membranous

## Data Availability

All data on which the manuscript was based, if not already included in its text, are available from the corresponding author on a reasonable request.

## Supplementary Materials

The following supporting information can be downloaded at: https://www.mdpi.com/article/10.3390/ijms26094114/s1.

## Abbreviations

The following abbreviations are used in this manuscript:

BCL9	B-cell CLL/lymphoma 9
ccRCC	Clear-cell renal cell carcinoma
CI	Confidence interval
COM	Calcium oxalate monohydrate
CPTAC	Clinical Proteomic Tumor Analysis Consortium
EPO	Erythropoietin
FFPE	Formalin-fixed paraffin-embedded
GO	Gene Ontology
HIF	Hypoxia-inducible factor
IHC	Immunohistochemistry
KSD	Kidney stone disease
LC-MS/MS	Liquid chromatography-tandem mass spectrometry
NAT	Normal adjacent tissue
OS	Overall survival
PFS	Progression-free survival
TAM	Tumor-associated macrophage
TGF	Transforming growth factor
TME	Tumor microenvironment
TPX2	Targeting Protein for Xenopus kinesin-like protein 2
VEGF	Vascular endothelial growth factor
